# The necessity of an observational study on the interactions between allergic history and citrus fruit intake for the prevention of pancreatic cancer

**DOI:** 10.4178/epih/e2015028

**Published:** 2015-06-16

**Authors:** Jong-Myon Bae

**Affiliations:** Department of Preventive Medicine, Jeju National University School of Medicine, Jeju, Korea

**Keywords:** Pancreatic neoplasms, Risk factors, Allergy and immunology, Citrus, Meta-analysis

## Abstract

While the main product of Jeju Island is citrus fruit and the prevalence of atopic dermatitis among the students who live there is relatively high, the incidence of pancreatic cancer is lowest in Korea. Systematic reviews reporting allergic history and intake of citrus fruit as protective factors against pancreatic cancer (PCC) were published in 2005 and 2008, respectively. Although there were discrepancies in the results of the subgroup analyses between case-control and cohort studies, it is necessary to evaluate an interaction effect between allergic history and intake of citrus fruits on PCC risk.

## INTRODUCTION

Pancreatic cancer is a primary site cancer that has the 4th highest cancer mortality in North America [1] and a representative malignant tumor with a less than 5% chance of having a 5-year survival rate [2]. Pancreatic cancer mortality was 5.6 per 100,000 among Korean populations in 2011, which was the 4th highest cancer mortality after lung cancer, liver cancer, and stomach cancer. It is the only primary site cancer to have shown no improvement in 5-year survival rate over the past 10 years [3]. Therefore, an analytical epidemiology study is desperately needed to reveal the factors responsible for either the increase or decrease in pancreatic cancer risk [4]. Known modifiable risk factors include smoking, diet, and medical history [5], among which the most significant factors, according to systematic reviews (SRs) were allergic history [6] and citrus fruit intake [7].

On the other hand, pancreatic cancer incidence in Jeju Island, which is localized in the lowest latitude among the Korean local governments, is markedly lower than that of other local governments [8]. By the way, Jeju Island is the main area of citrus fruit production, and it is the area with the highest atopic dermatitis prevalence among elementary, middle, and high school students [9]. Considering these health-environmental facts, the present study was performed to investigate if it was possible to propose a hypothesis that the interactions between citrus fruit intake and allergic history would lower pancreatic cancer incidence.

## BODY

### Inhibitory effects of allergic history

In the SR on the effects of allergic history on pancreatic cancer published by Gandini et al. [6] in 2005, the meta-analysis of 10 case-control studies (CCS) and 4 cohort studies (COS) resulted in the inhibition of pancreatic cancer incidence with 0.82 in total summary relative risk (sRR) and 0.68 to 0.99 in 95% confidence intervals (CIs). However, none of the 4 COS results were statistically significant [10-13].

**Figure 1.**
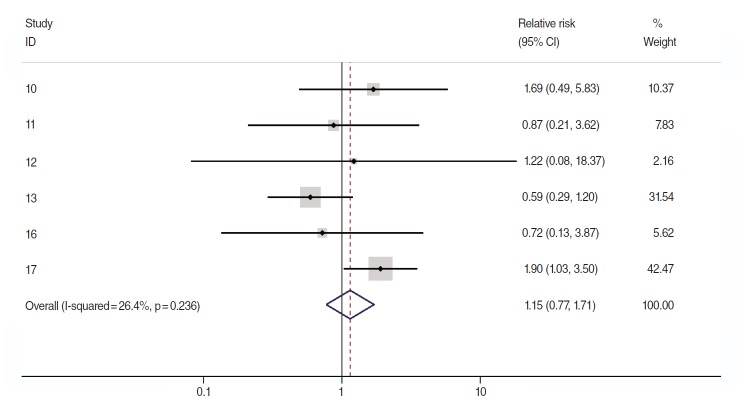
The forest plot of using a fixed-effects summary estimates in 6 cohort articles evaluating association between allergic history and pancreatic cancer risk. ID, reference number; CI, confidence interval.

**Figure 2.**
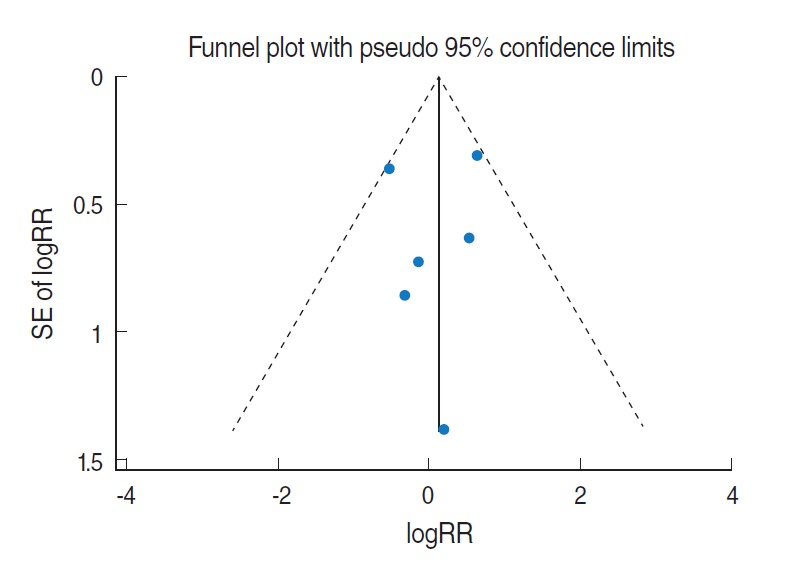
A funnel plot using fixed-effects summary estimates in 6 cohort articles evaluating the association between allergic history and pancreatic cancer risk. logRR, log relative risk; SE of logRR, standard error of log relative risk.

Related citation lists for each of the 4 COSs were made using the “related citations” function provided by PubMed (National Library of Medicine, US), followed by manual searches [14,15], which led to additional discoveries of two COS articles published in 2005 [16,17]. Nevertheless, there have been no COSs among articles published after 2006. Figure 1 is a forest plot that was obtained through meta-analysis using logarithmic relative risks (logRR) of a total of 6 target articles and their standard errors (SElogRR). The sRR value applied to the fixed effects model at 26.4% of the I-square [18] value representing degree of heterogeneity was 1.15 (95% CI, 0.77 to 1.71), which was not statistically significant. Begg’s test resulted in a p-value of 0.71, indicating no effect by a small-scale study, and there was no asymmetry effect on the corresponding funnel plot (Figure 2).

### Inhibitory effects of citrus fruit intake

In the SR on the effect of citrus fruit intake on pancreatic cancer performed by Bae et al. in 2009 [7], the meta-analysis with 4 CCSs and 5 COSs found inhibition effects of pancreatic cancer incidence (sRR, 0.83; 95% CI, 0.70 to 0.98). However, a subgroup analysis on the results of 5 COSs resulted in a sRR of 0.97 (95% CI, 0.86 to 1.10), which was not statistically significant.

Related citation lists for each of the 5 COSs [19-23] were made using the “related citations” function in PubMed, followed by manual searches [14,15], which led to the finding of 6 additional COS articles [24-29]. By the way, it was found that the same cohort was used in the articles by Stolzenberg-Solomon et al. [20] and Bobe et al. [24], so Bobe et al.’s 2008 article [24] was selected for an analysis. In addition, since the cohorts described in the articles by Shigihara et al. [28] and Li et al. [29] were also the same, so Shigihara et al.’s 2014 article [28] was selected. Moreover, the article by Coughlin et al. [19] concerning cancer mortality was excluded. Therefore, a total of 8 articles [21-28] were used for the meta-analysis.

**Figure 3.**
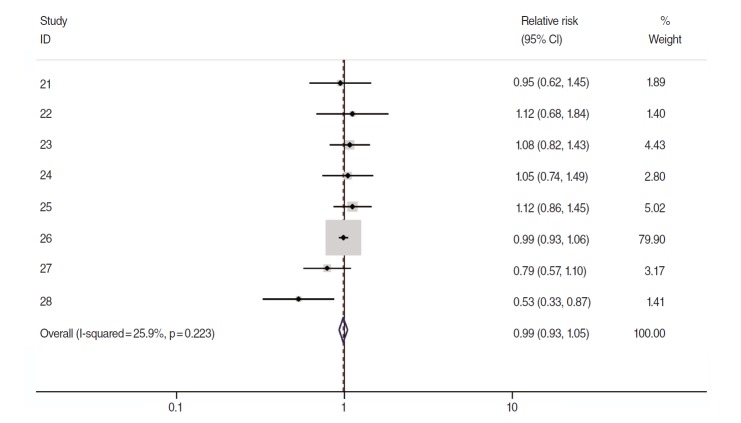
A forest plot using fixed-effects summary estimates in 8 cohort articles evaluating the association between intake of citrus fruits and pancreatic cancer risk. ID, reference number; CI, confidence interval.

**Figure 4.**
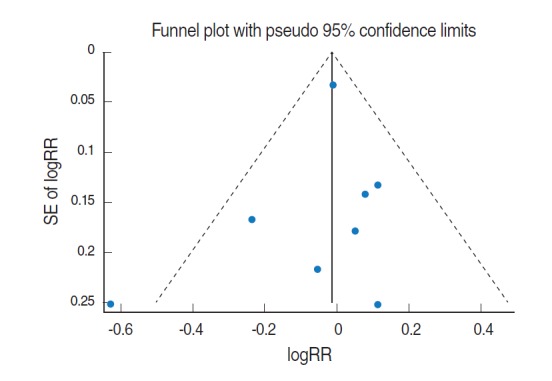
A funnel plot using fixed-effects summary estimates in 8 cohort articles evaluating the association between intake of citrus fruits and pancreatic cancer risk. logRR, log relative risk; SE of logRR, standard error of log relative risk.

Of these 8, sRR obtained from the meta-analysis of the results by gender and 95% CI were applied to the two articles [21,28] that presented RR by male/female. The resulting forest plot is shown in Figure 3. The sRR value applied to the fixed effects model at 25.9% of the I-squared [18] value was 0.99 (95% CI, 0.93 to 1.05), indicating it was not statistically significant. Begg’s test resulted in a *p*-value of 0.45, indicating no effect for the small-scale study, and there was no asymmetry effect on the corresponding funnel plot (Figure 4).

## DISCUSSION

According to the SR results in the COSs, there were no statistically significant results in allergic history and citrus fruit intake, respectively. When subgroup analysis results were contradictory between CCS and COS, it is reasonable to accept the SR result of the COS, rather than the CCS, due to the level of evidence [30].

However, when an analytical epidemiology study to reveal causality is performed on rare diseases with a low incidence like pancreatic cancer, a CCS study design is recommended, because COS needs a large group of participants. On the contrary, since CCS has a problem with recall bias risk on history of exposure [31], a pooled analysis study was recently performed with a larger number of research participants. In other words, after the meta-analysis report on citrus fruit [7], there was no statistically significant result found in a pooled analysis with 14 cohorts [26], while some experimental studies on citrus fruit components were published [4]. After the meta-analysis report on allergic history in 2005 [6], a pooled analysis that was performed with 10 CCSs resulted in a reduction of incidence risk [32]. However, there has been no report of pooled analysis with cohorts.

Of course, there is still a chance that measurement error in the history of exposure will persist, even in COS. For example, epidemiologic studies mostly depend on self-reported responses for allergic history and diet intake levels [26,31]. Furthermore, methodological limitations, such as a short of follow-up period and follow-up loss make it difficult to reveal causality [33]. In addition, since exposure levels applied to meta-analysis are relative levels, depending on distribution of intake amount it is hard to secure consistency between cohorts in the exposure level.

For now, SR of CCS results have consistently reported a reduced cancer risk, so that expert reviews have suggested that citrus fruit intake [4] and allergic history [31,32] had inhibitory effects on pancreatic cancer incidence. Therefore, it is necessary to investigate the hypothesis concerning the interactional effect of allergic history and citrus fruit intake, considering the pancreatic cancer incidence rate in Jeju Island. To date, there has been no analytical epidemiological study on the interaction between allergic history and citrus fruit intake in their inhibitory effects on pancreatic cancer, either inside or outside of Korea. Thus, I propose to perform an epidemiologic study on these interactional effect with cohorts securing both explanatory variables.
