# Supplementary material for: The necessity of an observational study on the interactions between allergic history and citrus fruit intake for the prevention of pancreatic cancer
Source: Epidemiol Health. 2015 Jun 16;37:e2015028. doi: 10.4178/epih/e2015028 (PMC4505181; doi:10.4178/epih/e2015028)
Supplement: Supplementary file 1 [file epih-37-e2015028-supplementary.pdf]

<Hypothesis>

췌장암 발생예방에 있어

알레르기 질병력과 감귤섭취의 상호작용 규명을 위한 관찰연구 필요성

It needs an observational study for Interaction of Allergic History and Citrus Fruit Intake as a Protective Factor in Pancreatic cancer

Abstract

While the main product of Jeju islands is citrus fruit and the prevalence of atopic dermatitis of dweller's students is relatively higher, the incidence of pancreatic cancer showed the lowest in Korea. The systematic reviews reporting allergic history and intake of citrus fruit as protective factors of pancreatic cancer (PCC) were published in 2005 and 2008, respectively. Although there were discrepancies in results of subgroup analyses between case-control studies and cohort studies, it is necessary to evaluate an interaction effect of allergic history and intake of citrus fruits in PCC risk.

Key words: Pancreatic neoplasms, Risk factor, Allergy and immunology, Citrus, Meta-analysis

서론

췌장암 (pancreatic cancer)은 북미에서 암사망률 순위 4위에 속하는 원발암 (primary site cancer)이며[1], 5년 생존률이 5% 미만인 대표적인 악성 종양이다[2]. 한국인에서도 2011년도 췌장암 사망률은 십만 명당 5.6명으로, 암사망률에 있어 폐암, 간암, 위암 다음으로 4위를 차지하며, 지난 10년간의 5년 생존율 개선이 없는 유일한 원발암이다[3]. 따라서 췌장암의 발생 위험 증가 혹은 감소 요인을 규명하는 분석역학 (analytical epidemiology) 연구가 절실히 필요하다[4]. 지금까지 알려진 개입 가능한 위험요인 (risk factors)들로는 흡연, 식이, 질병력 등이 있으며[5], 이중에서 체계적 고찰 (systematic reviews, SR)을 수행하여 유의미한 (significant) 연구결과를 확인한 요인으로는 알레르기 질병력[6]과 감귤 섭취[7]가 있다.

한편 대한민국의 지방자치단체 중 가장 낮은 위도에 위치한 제주도는 전국에 비하여 췌장암 발생률이 현저하게 낮다[8]. 그런데, 제주도는 감귤의 주요 생산지이면서, 초중고등학생에 있어 아토피 피부염 수진률이 전국에서 가장 높은 지역으로 알려져 있다[9]. 이런 보건환경적인 사실들을 감안할 때, 저자는 감귤섭취와 알레르기 질병력의 상호작용이 췌장암 발생률을 낮춘다는 가설을 제시할 수 있는가를 검토해 보았다.

본문

가. 알레르기 질병력의 발생억제 효과

2005년도 발표된 Gandini et al. [6]이 수행한 췌장암에 있어 알레르기 질병력의 영향에 대한 SR에서, 10편의 환자-대조군 연구 (case-control studies, CCS)와 4편의 코호트 연구 (cohort studies, COS) 의 메타분석 결과는 총 상대위험도 (summary relative risk, sRR) 0.82, 95% 신뢰구간 (confidence intervals, CIs) 0.68-0.99로 췌장암 발생을 억제하는 것으로 나왔다. 그런데, 4편의 COS 각각의 결과들[10-13]은 통계적 유의성을 보이지 않았다.

PubMed (National Library of Medicine, US) 에서 제공하는 'Related citations' 기능을 활용하여 4편의 COS 각각의 검색 목록을 만든 다음, 일일이 손으로 검색하였다[14,15]. 그 결과, 2005년도에 발표된 두 편의 COS 논문[16,17]을 추가로 확보하였다. 반면 2006년도 이후 발표된 코호트 연구는 찾을 수 없었다. Fig 1은 총 6편의 선정대상 논문들의 로그화 상대위험도 (logRR) 과 이의 표준오차 (SElogRR)을 활용하여 메타분석결과로 얻어낸 forest plot 이다. 이질성 수준을 나타내는 I-square [18] 수치 26.4%에서 고정효과모형을 적용한 sRR은 1.15 (95% CI: 0.77-1.71)로 통계적 유의성이 없었다. Begg's test 수행결과 p-값은 0.71로 소규모 연구에 영향을 받지 않았으며, 해당 funnel plot 상 대칭성을 해치지는 않았다 (Fig 2).

나. 감귤섭취의 발생억제효과

2009년도 발표된 Bae et al. [7]이 수행한 췌장암에 있어 감귤섭취의 영향에 대한 SR에서도 4편의 CCS와 5편의 COS를 합친 메타분석 결과는 암발생 감소효과가 있는 것으로

나왔다 (sRR=0.83, 95% CI, 0.70-0.98). 그러나 5 편의 COS 연구결과에 대한 하부군 분석 (subgroup analysis)에서 sRR 은 0.97 (95% CI, 0.86-1.10)으로 통계적 유의성이 없었다.

PubMed의 'Related citations' 기능을 활용하여 5편의 COS [19-23] 각각의 검색 목록을 만든 다음, 일일이 손으로 검색하였다[14,15]. 그 결과, 6편의 COS 논문[24-29]을 추가로 확보하였다. 그런데 Stolzenberg-Solomon et al. [20] 논문과 Bobe et al. [24] 논문의 코호트는 동일한 것으로 확인되어, 2008년도 발간된 논문[24]을 분석 대상으로 선정하였다. 또한 Shigihara et al. [28] 논문과 Lie et al. [29] 논문의 코호트 구성원도 동일하여, 이중 2014년도에 발간된 논문[28]을 선정하였다. 또한 Coughlin et al. [19] 논문은 암사망률을 다룬 논문이었다. 따라서 총 8편[21-28]을 메타분석의 대상으로 삼았다

이중 남녀별로 RR을 제시한 논문 2 편 [21,28]은 성별 결과를 메타분석하여 얻은 sRR과 95% CI을 적용하였다. Fig. 3은 그 결과로 얻어낸 forest plot이다. 이질성 수준을 나타내는 I-squared [18] 수치 25.9%에서 고정효과모형을 적용한 sRR 0.99 (95% CI: 0.93-1.05)로 통계적 유의성이 없었다. Begg's test 결과 p-값은 0.45으로 소규모 연구에 영향을 받지 않았으며, 해당 funnel plot 상 대칭성을 해치지는 않았다 (Fig 4).

결론 및 제언

알레르기 질병력과 감귤 섭취 모두 COS 연구들의 SR 결과에서 통계적 유의성을 확보하지 않았다. CCS와 COS 로 나누어 하부군 분석을 한 결과가 서로 상반되는 경우는, 근거의 계층에 따라 CCS보다는 COS의 SR 결과를 우선 수용하는 것이 타당할 것이다[30].

그렇지만 인과성 규명을 위한 분석역학연구에서 췌장암처럼 발생률이 낮은 희귀 질환 (rare diseases)을 다룰 경우는 COS 연구 수행시 대규모의 관찰 대상자를 확보해야 하는 만큼, CCS 연구설계를 우선 추천한다. 반면 CCS인 경우 노출력에 대한 회상오류 (recall bias)가 개입될 수 있다는 문제 때문에[31], 최근에는 연구 대상자 규모를 증대시킨 통합분석 (pooled analysis) 연구를 수행하고 있다. 즉, 감귤에 있어 메타분석결과[7]가 보고된 이후, 감귤 성분에 대한 실험실적 연구들[4]이 발표되는 한편, 14개의 코호트를 모은 통합분석 결과는 통계적 유의성이 없었다[26]. 알레르기 질병력의 메타분석[6]이 2005년 보고된 이후, 10개의 CCS를 모아서 통합분석 (pooled analysis)를 시행했을 때, 발생위험 감소효과가 있는 것으로 나왔다[32]. 그러나 코호트를 모은 통합분석결과는 아직 발표된 것이 없다.

물론, COS 연구라 해도 노출력에 대한 측정 오류 (measurement error)의 가능성이 여전히 있다. 예를 들어 역학연구에서 알레르기 질병력과 식이 섭취 수준을 대부분 자기보고식 (self-reported) 응답에 의존하고 있으며[26,31], 추적 기간의 부족과 추적 소실 (follow-up loss) 등의 방법론적 한계는 인과성 규명을 어렵게 한다[33]. 또한 메타분석에 적용하는 노출수준은 섭취량의 분포에 따른 상대적 수준을 다루기 때문에, 노출수준에 있어 코호트 간에 일관성을 확보하기가 어렵다.

현재로는 CCS 연구 결과들의 SR은 일관되게 암발생 위험을 낮추는 것으로 보고되면서, 전문가 종설 (expert review)에서는 감귤 섭취[4]와 알레르기 질병력[31,32]은 췌장암의 발생 억제 효과가 있다고 해석하고 있다. 그렇다면 제주도의 췌장암 발생현황을 고려할 때 알레르기 질병력과 감귤 섭취의 상호효과에 대한 가설 규명은 필요하다고 볼 수 있다. 지금까지 국내외를 통틀어 알레르기 질병력과 감귤 섭취의 상호작용에 따른 췌장암 억제효과에 대한 분석역학 연구는 아직 보지 못하였다. 두 설명변수를 모두 확보한 코호트에서 이의 상호효과에 대한 분석을 시도하기를 제안한다.

감사의 글

이 논문은 2015학년도 제주대학교 학술진흥연구비 지원사업에 의하여 연구되었음

References

1. Siegel R, Naishadham D, Jemal A. Cancer statistics, 2012. CA Cancer J Clin 2012;62:10-29.
2. Klapman J, Malafa MP. Early detection of pancreatic cancer: why, who, and how to screen. Cancer Control 2008;15:280-287.
3. Jung KW, Won YJ, Kong HJ, Oh CM, Lee DH, Lee JS. Cancer statistics in Korea: incidence, mortality, survival, and prevalence in 2011. Cancer Res Treat 2014;46:109-123.
4. Pericleous M, Rossi RE, Mandair D, Whyand T, Caplin ME. Nutrition and pancreatic cancer. Anticancer Res 2014;34:9-21.
5. Lowenfels AB, Maisonneuve P. Epidemiology and risk factors for pancreatic cancer. Best Pract Res Clin Gastroenterol 2006;20:197-209.
6. Gandini S, Lowenfels AB, Jaffee EM, Armstrong TD, Maisonneuve P. Allergies and the risk of pancreatic cancer: a meta-analysis with review of epidemiology and biological mechanisms. Cancer Epidemiol Biomarkers Prev. 2005;14:1908-1916.
7. Bae JM, Lee EJ, Guyatt G. Citrus fruit intake and pancreatic cancer risk: a quantitative systematic review. Pancreas 2009;38:168-174.
8. Bae JM. Explaining cancer incidence in the Jejudo population. J Prev Med Public Health 2009;42:67-72. (Korean)
9. Bae JM, Shin KS. Estimating the prevalence of atopic dermatitis in school students of Jejudo, Korea. J Prev Med Public Health 2009;42:171-176. (Korean)
10. McWhorter WP. Allergy and risk of cancer. A prospective study using NHANESI followup data. Cancer 1988;62:451-455.
11. Mills PK, Beeson WL, Abbey DE, Fraser GE, Phillips RL. Dietary habits and past medical history as related to fatal pancreas cancer risk among Adventists. Cancer 1988;61:2578-2585.
12. Eriksson NE, Holmén A, Högstedt B, Mikoczy Z, Hagmar L. A prospective study of cancer incidence in a cohort examined for allergy. Allergy 1995;50:718-722.
13. Stolzenberg-Solomon RZ, Pietinen P, Taylor PR, Virtamo J, Albanes D. A prospective study of medical conditions, anthropometry, physical activity, and pancreatic cancer in male smokers (Finland). Cancer Causes Control 2002;13:417-426.
14. Bae JM. Narrative reviews. Epidemiol Health 2014;36:e2014018.
15. Bae JM. Human papillomavirus 16 infection as a potential risk factor for prostate cancer: an adaptive meta-analysis. Epidemiol Health 2015;37:e2015005.
16. Eriksson NE, Mikoczy Z, Hagmar L. Cancer incidence in 13811 patients skin tested for allergy. J Investig

Allergol Clin Immunol 2005;15:161-166.

17. Hagströmer L, Ye W, Nyrén O, Emtestam L. Incidence of cancer among patients with atopic dermatitis. Arch Dermatol 2005;141:1123-1127.
18. Higgins JP, Thompson SG. Quantifying heterogeneity in a meta-analysis. Stat Med 2002;21:1539-1558.
19. Coughlin SS, Calle EE, Patel AV, Thun MJ. Predictors of pancreatic cancer mortality among a large cohort of United States adults. Cancer Causes Control 2000;11:915-923.
20. Stolzenberg-Solomon RZ, Pietinen P, Taylor PR, Virtamo J, Albanes D. Prospective study of diet and pancreatic cancer in male smokers. Am J Epidemiol 2002;155:783-792.
21. Lin Y, Kikuchi S, Tamakoshi A, Yagyu K, Obata Y, Inaba Y, et al. Dietary habits and pancreatic cancer risk in a cohort of middle-aged and elderly Japanese. Nutr Cancer 2006;56:40-49.
22. Larsson SC, Håkansson N, Näslund I, Bergkvist L, Wolk A. Fruit and vegetable consumption in relation to pancreatic cancer risk: a prospective study. Cancer Epidemiol Biomarkers Prev. 2006;15:301-305.
23. Nöthlings U, Murphy SP, Wilkens LR, Henderson BE, Kolonel LN. Dietary glycemic load, added sugars, and carbohydrates as risk factors for pancreatic cancer: the Multiethnic Cohort Study. Am J Clin Nutr 2007;86:1495-1501.
24. Bobe G, Weinstein SJ, Albanes D, Hirvonen T, Ashby J, Taylor PR, et al. Flavonoid intake and risk of pancreatic cancer in male smokers (Finland). Cancer Epidemiol Biomarkers Prev 2008;17:553-562.
25. Vrieling A, Verhage BA, van Duijnhoven FJ, Jenab M, Overvad K, Tjønneland A, et al. Fruit and vegetable consumption and pancreatic cancer risk in the European Prospective Investigation into Cancer and Nutrition. Int J Cancer 2009;124:1926-1934.
26. Koushik A, Spiegelman D, Albanes D, Anderson KE, Bernstein L, van den Brandt PA, et al. Intake of fruits and vegetables and risk of pancreatic cancer in a pooled analysis of 14 cohort studies. Am J Epidemiol 2012;176;373-386.
27. Heinen MM, Verhage BA, Goldbohm RA, van den Brandt PA. Intake of vegetables, fruits, carotenoids and vitamins C and E and pancreatic cancer risk in The Netherlands Cohort Study. Int J Cancer 2012;130:147-158.
28. Shigihara M, Obara T, Nagai M, Sugawara Y, Watanabe T, Kakizaki M, et al. Consumption of fruits, vegetables, and seaweeds (sea vegetables) and pancreatic cancer risk: the Ohsaki Cohort Study. Cancer Epidemiol 2014;38:129-136.
29. Li WQ, Kuriyama S, Li Q, Nagai M, Hozawa A, Nishino Y, et al. Citrus consumption and cancer incidence: the Ohsaki cohort study. Int J Cancer 2010;127:1913-1922.
30. Health Sciences Library, UNC-Chapel Hill. Type of Questions and studies. [cited 2015 Apr 13].

Available from: http://www.hsl.unc.edu/Services/Tutorials/EBM/Supplements/QuestionSupplement.htm.

31. Turner MC, Chen Y, Krewski D, Ghadirian P. An overview of the association between allergy and cancer. Int J Cancer 2006;118:3124-3132.

32. Rittmeyer D, Lorentz A. Relationship between allergy and cancer: an overview. Int Arch Allergy Immunol 2012;159:216-225.

33. Olson SH, Hsu M, Satagopan JM, Maisonneuve P, Silverman DT, Lucenteforte E, et al. Allergies and risk of pancreatic cancer: a pooled analysis from the Pancreatic Cancer Case-Control Consortium. Am J Epidemiol 2013;178:691-700.
